# Toward augmented reality in laparoscopic liver surgery using electromagnetic tracking: a clinical feasibility study

**DOI:** 10.1007/s00464-026-12741-5

**Published:** 2026-03-18

**Authors:** Karin A. Olthof, Lisanne P. J. Venix, Maaike Pruijt, Anne G. den Hartog, Niels F. M. Kok, Theo J. M. Ruers, Matteo Fusaglia, Koert F. D. Kuhlmann

**Affiliations:** 1 Department of Surgical Oncology, Netherlands Cancer Institute, Plesmanlaan 121, 1066CX Amsterdam, The Netherlands; 2 Faculty of Science and Technology (TNW), Nanobiophysics Group (NBP), University of Twente, Drienerlolaan 5, 7522 NB Enschede, The Netherlands

**Keywords:** Laparoscopic liver surgery, Augmented reality, Surgical navigation, Image-guided surgery

## Abstract

**Background:**

Augmented reality (AR) has the potential to enhance intraoperative visualization, orientation and precision in laparoscopic liver surgery by combining preoperative imaging data with surgical reality. Accurate registration and motion compensation remain significant challenges for clinical implementation. This study aims to translate electromagnetic (EM)-tracked navigation to the laparoscopic setting, incorporate AR and assess clinical feasibility and accuracy.

**Methods:**

The open navigation workflow was adapted by implementing EM tracking for a pointer, laparoscopic ultrasound (LUS) probe, and laparoscope. Four AR visualization modes were developed and tested ex vivo on a liver phantom; seven hepatobiliary surgeons rated essential anatomical structures and the preferred display modes. A prospective feasibility study was conducted in patients undergoing laparoscopic liver resection. An EM sensor was attached to the liver surface, landmark-based registration was performed using tracked LUS and pointer, and AR overlays were displayed intraoperatively. Outcomes included technical feasibility of the surgical workflow, registration accuracy and intraoperative AR performance.

**Results:**

The translation of the EM navigation workflow to the laparoscopic setting yielded robust tracking performance across all laparoscopic instruments. Surgeons rated the tumor, hepatic veins, and portal veins as the most important structures for AR visualization and preferred displays emphasizing a resection plane with target structures and applying depth shading to improve depth perception. The complete workflow (i.e., sensor attachment, registration and AR visualization) was achieved in 13 of 14 procedures without device-related complications. Mean target registration error was 6.3 ± 3.8 mm and registration took an average of 11 ± 7 min.

**Conclusions:**

AR navigation during laparoscopic liver surgery using EM tracking is feasible and enables organ tracking including local motion compensation. This workflow supports intraoperative orientation and tumor localization in minimally invasive resections potentially improving clinical outcome.

**Supplementary Information:**

The online version contains supplementary material available at 10.1007/s00464-026-12741-5.

Minimally invasive liver surgery is adopted as the current state-of-the-art for a significant part of procedures, as it is associated with reduced postoperative morbidity, less pain, and shorter hospital stay compared to open surgery [1–3]. However, the minimally invasive approach is constrained by a restricted field of view, loss of tactile feedback, and reduced intraoperative orientation, limiting its application in more complex procedures [4, 5]. Augmented reality (AR) is an image-guidance technology which holds the potential to improve the accuracy and safety of laparoscopic surgery. As AR overlays virtual 3D models of the patient’s anatomy onto the laparoscopic video, it allows surgeons to identify tumors and vital surrounding structures.

While promising, AR has not yet been widely adopted in liver surgery due to several technical challenges. Achieving and maintaining accurate registration between preoperative models and the intraoperative situation is particularly difficult [6]. Furthermore, the integration of tracking hardware and the need to compensate for organ deformation remain significant barriers to routine clinical implementation. Most existing approaches achieve registration either through manual alignment or by sampling surface landmarks [7–12]. Manual alignment is inherently subjective and challenging in three dimensions, while exact localization of surface landmarks on the smooth hepatic surface is error-prone. Semi-automatic registration methods based on 3D organ surface reconstruction from laparoscopic video remain highly susceptible to image noise from reflections, blood, and neighboring organs [13–16]. As a result, accuracy and robustness have not yet reached the level required for reliable clinical use. Maintaining an accurate alignment throughout the procedure presents another challenge. Intraoperative liver motion, caused by respiration and surgical manipulation, disrupts registration accuracy unless actively compensated.

In addition to accurate registration and motion compensation, the clinical utility of AR strongly depends on the manner in which anatomical information is presented to the surgeon. Effective visualization strategies must provide clear, relevant guidance while avoiding any obstruction or distraction within the operative field [8]. Consequently, evaluation and optimization of AR interface designs are essential to support safe, efficient and intuitive decision-making in minimally invasive liver surgery.

In previous work, we developed a surgical navigation system for open liver surgery incorporating liver tracking [17]. In this workflow, an electromagnetic (EM) sensor is attached onto the liver surface in proximity to the target lesion. This sensor captured and compensated local organ motion caused by respiration and surgical manipulation. Motion tracking was supported by a robust registration method, in which a tracked ultrasound probe was used to sample internal landmarks located in the proximity of the targeted lesion. By providing an accurate overlay of preoperative liver models onto the live ultrasound image, this approach improved intraoperative tumor localization [18, 19]. As it allows for accurate treatment of disappearing liver metastases, it is now standard-of-care in our institute.

The aim of this study was to translate these findings to the laparoscopic setting, thereby combining benefits of navigation with those of minimally invasive procedures. Building on our previous research where 3D models were superimposed onto ultrasound images, the proposed system explores direct overlay of anatomical models onto the laparoscopic video. This translation required adapting the navigation workflow for laparoscopic use, developing and evaluating AR interfaces through ex vivo testing, and ultimately assessing the clinical feasibility of the complete workflow during laparoscopic liver resection.

## Materials and methods

### Translation from open to laparoscopic EM navigation

The navigation workflow previously developed in [17–19] for open liver surgery was adapted for laparoscopic use. Instrument tracking was performed using the Aurora® V2 electromagnetic (EM) planar field generator (Northern Digital Inc., Waterloo, Canada). Three instruments were equipped with EM sensors: a laparoscopic pointer, a laparoscopic ultrasound (LUS) probe (I12C4f (9066), BK Medical, Denmark), and a 30° laparoscope (Endoeye 30° HD, Olympus, Japan). EM sensors used in this study were commercial products (Northern Digital Inc., Waterloo, Canada).

High-resolution 3D scans of the standard laparoscopic instruments (ultrasound probe and laparoscope) were acquired to map their exact geometry (Artec 3D, Luxembourg, Luxembourg). Using these scans, we designed and 3D-printed form-fitting adapters that house the EM sensors. This ensures a unique, stable fit for each instrument, which is critical for accurate calibration. The design of the adapters was performed using SolidWorks 2022 (Dassault Systèmes, Vélizy-Villacoublay, France) and 3D printed using a Formlabs Form 3B + stereolithography printer (Formlabs Inc., Somerville, MA, USA). The 3D-printed adapters are non-sterile components; they are attached to the instruments by the technician and covered with a sterile surgical drape prior to the procedure. Conversely, the liver sensor and the pointer are sterile devices that can be handled directly by the surgeon. This workflow ensures that standard hospital equipment can be tracked without permanent modification or compromised sterility.

**Fig. 1:**
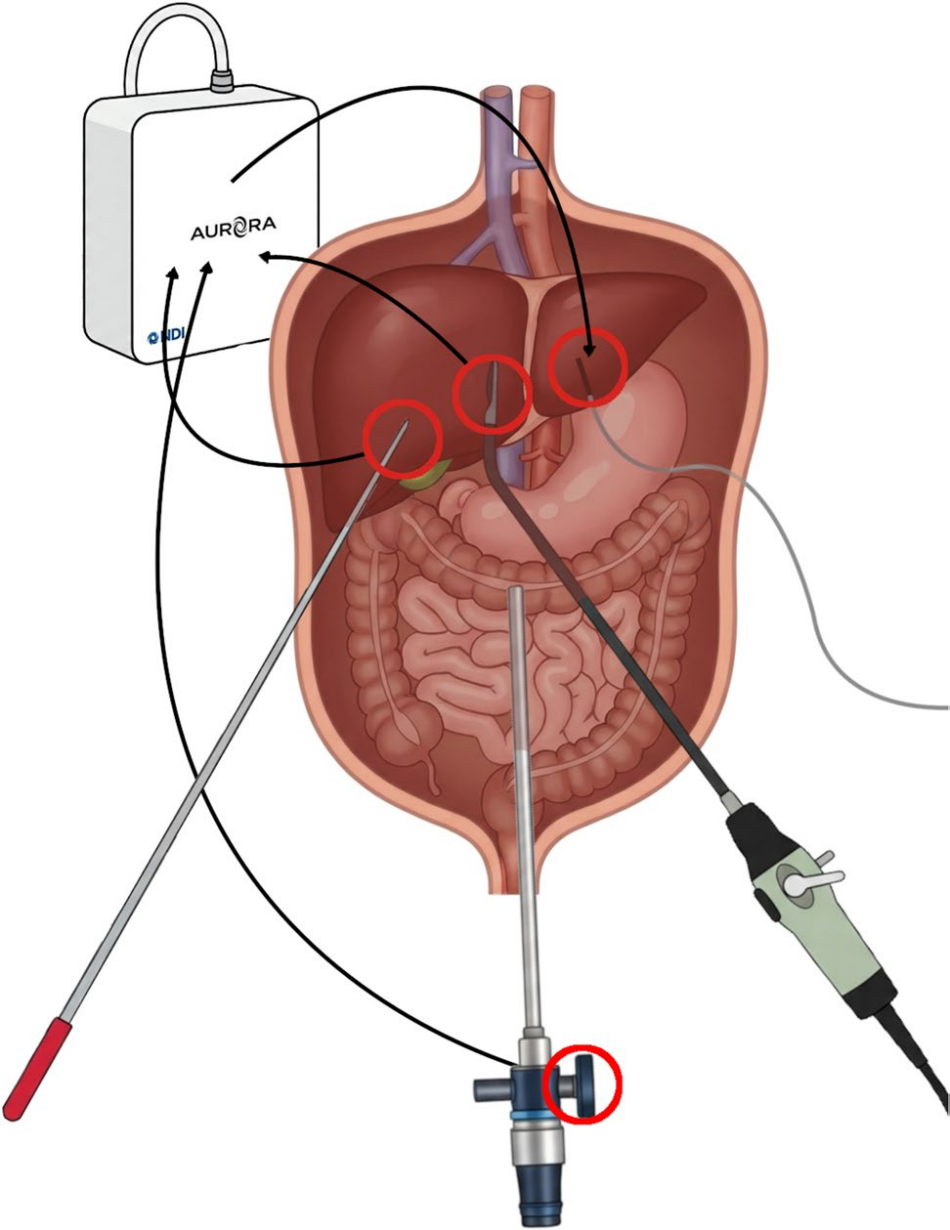
Schematic overview of the tracked instruments, including the laparoscope, laparoscopic ultrasound probe, pointer, and liver sensor with sensor positions denoted by red circles

The laparoscopic pointer was constructed from stainless-steel (316L) tubing with an EM sensor embedded at its distal tip. The LUS adapter was designed to create a unique alignment between the sensor and imaging plane while fitting through a standard 12-mm trocar. The laparoscope adapter positioned the EM sensor externally around the trocar sleeve to maintain proximity to the operative field and to avoid EM interference with the internal electronics of the laparoscope, as described by Liu et al. [20]. All tracked components were designed to remain within the effective 50 × 50 × 50 cm operational tracking volume of the field generator when used clinically (Fig. 1).

Preoperative calibration of the LUS probe was performed with an EM-tracked pointer, and the laparoscope was calibrated using Zhang’s method [21, 22]. The instrument 3D models, together with their calibrations, were integrated into the CustusX version 18.04 (SINTEF, Trondheim, Norway) navigation software. To enable AR navigation, the laparoscopic video feed was incorporated into the platform by routing the Olympus video output through a frame grabber (Epiphan DVI2USB 3.0) and importing it into the navigation system. A virtual environment was developed in Unity 3D (Unity Technologies, San Francisco, USA), allowing real-time integration of 3D instrument models, tracking data, and laparoscopic video.

The workflow for liver-sensor placement, landmark acquisition, and registration is described in the Clinical feasibility study section.

### AR visualizations and ex vivo evaluation

Four AR display modes were designed in Unity 3D to optimize anatomical visualization: (a) complete 3D model showing internal structures and liver surface; (b) the planned resection plane and vascular branches supplying the resected segment; (c) 3D model limited to a circular region centered on the laparoscopic image to reduce visual clutter; and (d) increased transparency of structures further away from the laparoscope to improve depth perception.

Ex vivo experiments of the four AR displays were performed using the IOUSFAN phantom (Kyoto Kagaku Co., Ltd, Kyoto, Japan). Video recordings of these experiments were presented to seven hepatobiliary surgeons from two hospitals. The surgeons were asked to indicate which hepatic structures they considered essential for AR display and which visualization modes they preferred for clinical use. Multiple preferences could be selected. The phantom study provided a general impression of overall preferred visualization modes and familiarized surgeons with the available options. Visualization preferences varied between surgeons, and were also influenced by the specific clinical task. Given the feasibility nature of the study, no standardized visualization mode was enforced in the clinical study. Instead, for each clinical procedure, the operating surgeon selected the preferred visualization mode based on prior experience with the phantom study and the anticipated intraoperative requirements.

### Clinical feasibility study

#### Patient inclusion

A prospective, single center feasibility study was conducted at the Netherlands Cancer Institute. Consecutive patients scheduled for laparoscopic resection of liver tumors between September 2023 and March 2025 were eligible for inclusion, irrespective of tumor type, size, or location. Exclusion criteria included patients with vanished lesions on intraoperative ultrasound. While this patient group represents a primary indication for image guidance, tumor visibility was strictly required in this study to serve as a ground truth for calculating the target registration error (TRE) and validating system accuracy. Patients with a pacemaker were also excluded because of potential interference with the EM field generator.

This study was conducted in accordance with the Declaration of Helsinki and was approved by the institutional medical ethics committee in August 2023 (NL65724.031.18) and written informed consent was obtained from all participants prior to surgery. Demographic characteristics and perioperative variables were recorded, including operative time, conversion to open surgery, postoperative complications, length of hospital stay, length of hospital stay and resection margin status (R0/R1).

#### Surgical workflow

**Fig. 2:**
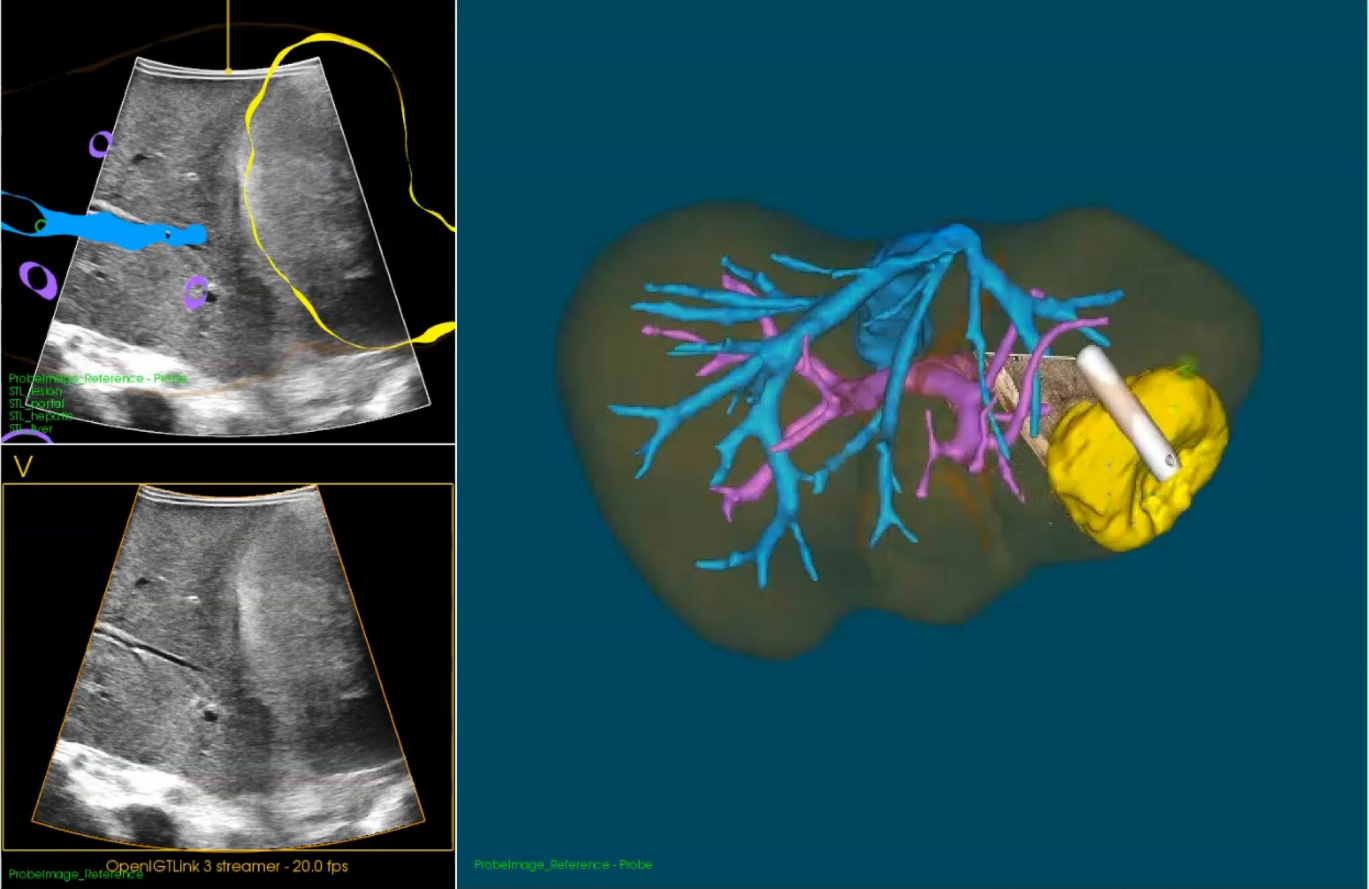
Navigation visualization with a 3D model of the liver with the parenchyma (brown), hepatic vein (blue), portal vein (purple) and tumor (yellow) on the right side and a cross-sectional overlay of the 3D model over the live ultrasound image on the top left. This allows for direct assessment of registration accuracy

3D models of the hepatic anatomy were generated preoperatively from diagnostic scans according to [18, 19]. Prior to each procedure, the preferred AR visualizations were discussed with the operating surgeon and adjusted accordingly for the specific case. The LUS probe, laparoscope, and field generator were covered with sterile drapes. To compensate for local movement due to respiration and surgical manipulation, an EM sensor was secured intraoperatively to the parenchyma adjacent to the target tumor using a laparoscopic glue applicator (Glubran®, GEM, Italy). The sensor was positioned within the planned resection planes, ensuring that it was removed together with the tumor. Registration of the preoperative three-dimensional (3D) liver model to the intraoperative anatomy was performed in CustusX. Corresponding anatomical landmarks near the target lesion were identified on the preoperative model and digitized intraoperatively using tracked instruments. Subsurface landmarks, such as vascular bifurcations and cysts, were sampled with the EM-tracked LUS probe, whereas surface landmarks, including ligament insertions, were digitized using the tracked laparoscopic pointer. Registration accuracy was verified by superimposing the 3D model onto the live ultrasound image and assessing correspondence of structures such as vessels and tumor (Fig. 2). To enable AR, the registration matrix was transferred to Unity.

#### Clinical evaluation

The primary outcome was feasibility of the complete navigation workflow, including sensor attachment, registration and AR visualization without technical errors. Secondary outcomes included registration accuracy, time required for individual study steps, and the intraoperative performance of augmented reality overlays. Registration accuracy was assessed using the target registration error (TRE), defined as the positional difference between the tumor location indicated by the navigation system and its intraoperative location. To obtain an intraoperative reference, the tracked LUS probe was used to acquire a sweep encompassing the entire tumor, from which a 3D ultrasound volume was reconstructed. The tumor was segmented from this volume and its center was used as the reference position. TRE was calculated as the Euclidean distance between this ultrasound-derived tumor center and the corresponding tumor center in the navigation system after registration. In addition, the fiducial registration error (FRE) was computed as the root-mean-square of the residual distances between all digitized anatomical landmarks (ranging from 3 to 6 landmarks per case) and their corresponding points on the preoperative model after registration. In addition, time was recorded for key steps of the workflow, including attachment of the liver sensor, registration and initializing of AR. In this feasibility study, AR visualization was restricted to observational use and not applied for surgical guidance.

## Results

### Translation from open to laparoscopic EM navigation

**Fig. 3:**
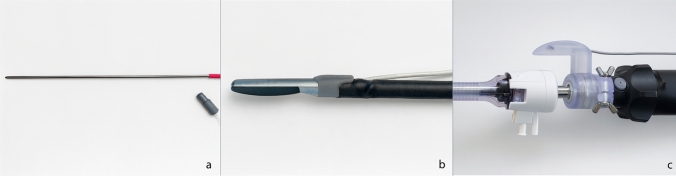
Tracked surgical instruments included **a** a laparoscopic pointer, **b** a laparoscopic ultrasound probe, and **c** a 30° laparoscope

The EM-tracked laparoscopic pointer and the 3D-printed adapters for the LUS probe and laparoscope are shown in Fig. 3. The LUS adapter allowed unrestricted use through 12-mm trocars without friction or loss of probe angulation. The laparoscope adapter enabled tracking of the position and orientation of its shaft, but did not capture the additional rotation of the optical system around its longitudinal axis, typical of laparoscopes with a 30°viewing angle; therefore, a fixed viewing angle was used in this feasibility study. The calibration of the LUS probe yielded a root-mean-square error (RMSE) of 0.97 mm across 48 calibration points. Laparoscope calibration achieved an average reprojection error of 0.27 pixels across 20 calibration images, consistent with previously reported accuracies [20]. All tracked instruments remained within the operational tracking volume during use, and no EM interference with laparoscope electronics was observed.

### Ex vivo AR evaluation

**Fig. 4:**
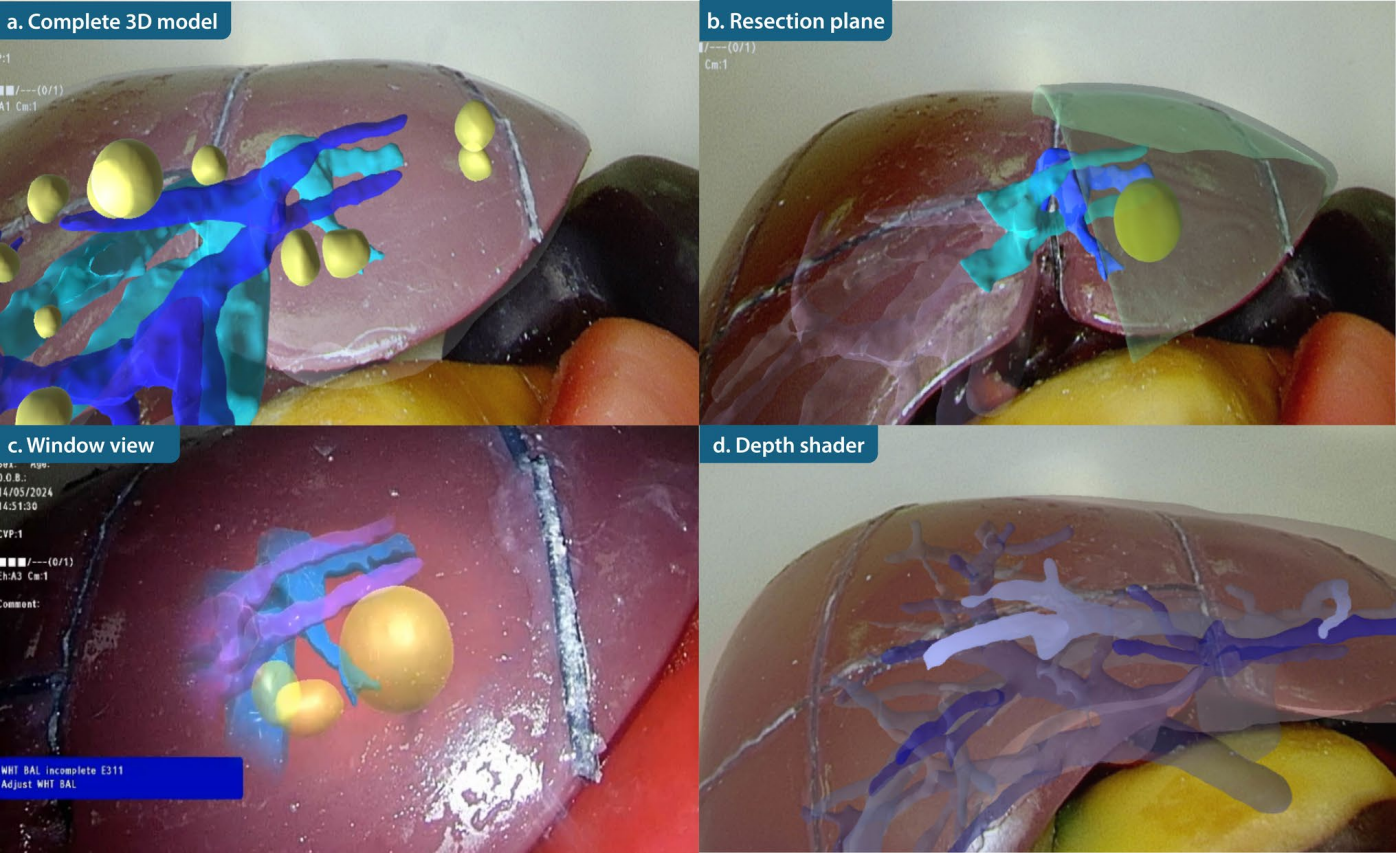
AR visualizations in a phantom setup: **a** complete 3D liver model overlay; **b** planned resection plane with vascular branches supplying the segment to be resected; **c** AR overlay restricted to the central laparoscopic field; **d** reduced opacity of structures closer to the laparoscope to enhance depth perception

**Fig. 5:**
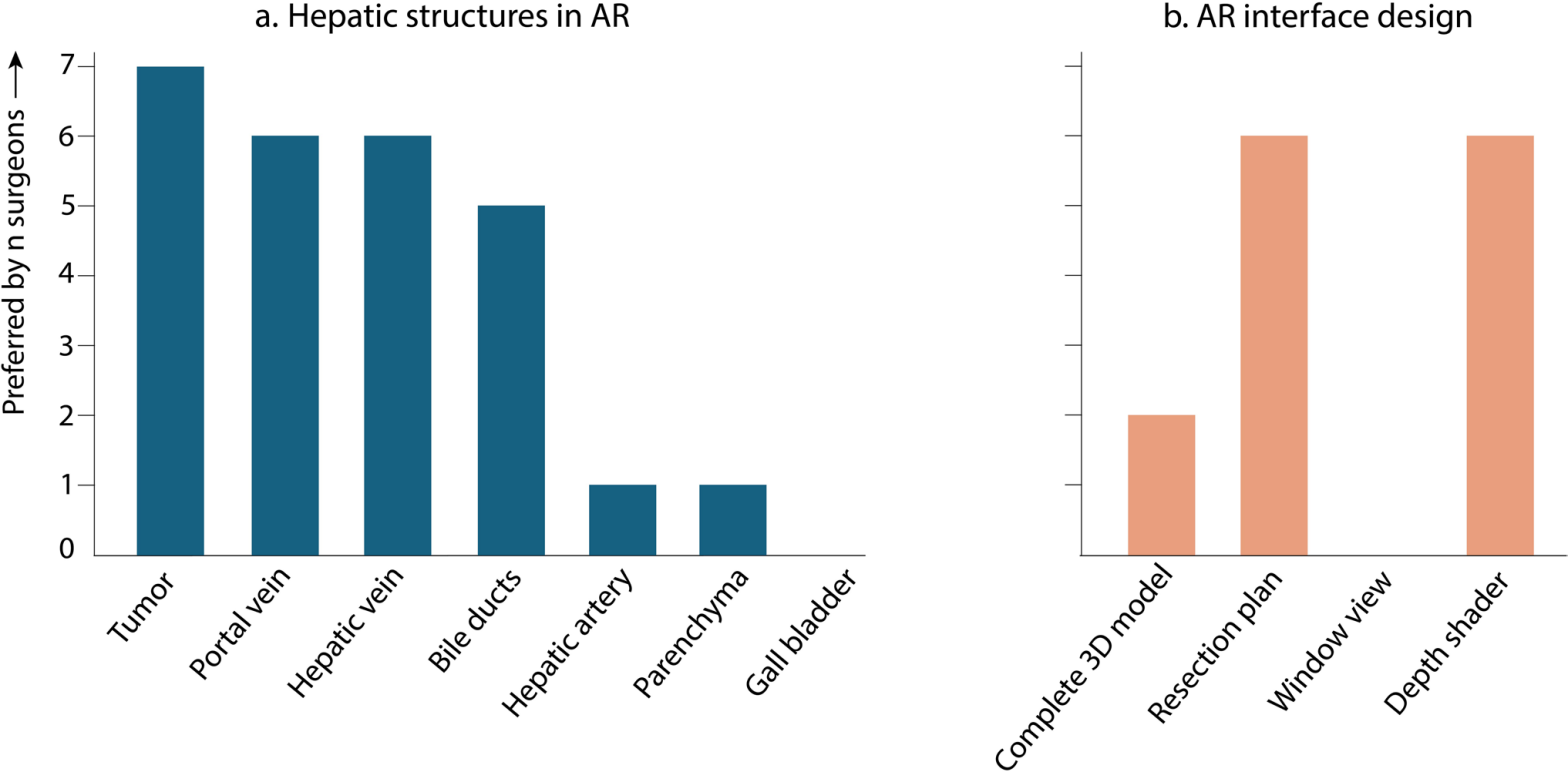
Preferences for AR visualization as rated by seven hepatobiliary surgeons with **a** the hepatic structures in AR, and **b** the AR interface design

Four AR visualizations were successfully implemented (Fig. 4). These were evaluated by 7 hepatobiliary surgeons from two centers. Among the hepatic structures, the tumor, portal vein, and hepatic vein were considered most important for intraoperative AR visualization, followed by the bile ducts (Fig. 5a). Arterial anatomy, the liver parenchyma, and the gall bladder were considered of lower priority. Most surgeons favored visualizations highlighting the resection plane and target structures to guide resection (*n* = 6) or applying a depth-shading effect to enhance spatial perception (*n* = 6). Projection of the complete liver model was selected less frequently (*n* = 2), as it could occlude the laparoscopic view and distract from the operative field (Fig. 5b). The window view, while reducing visual clutter, was not selected because it limited the surgical perspective and did not provide continuous AR visualization of the tumor and target structures. The visualization modes were incorporated into the AR displays used in subsequent clinical implementation.

### Clinical feasibility study

**Fig. 6:**
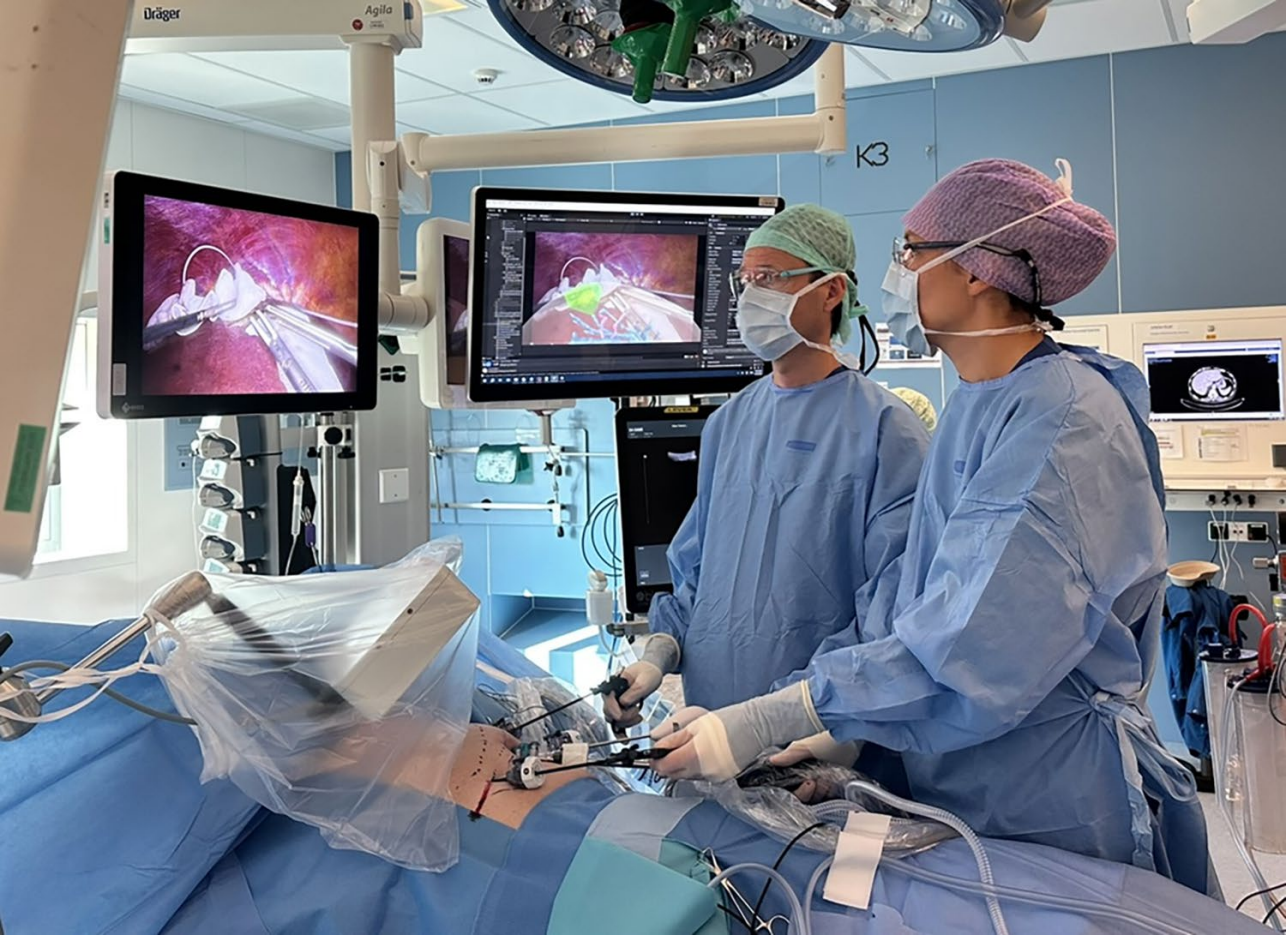
Surgical navigation setup. The EM field generator localizes the tracked surgical instruments and provides real-time navigation. Surgeons are provided with standard (left) and augmented reality (right) laparoscopic views

**Fig. 7:**
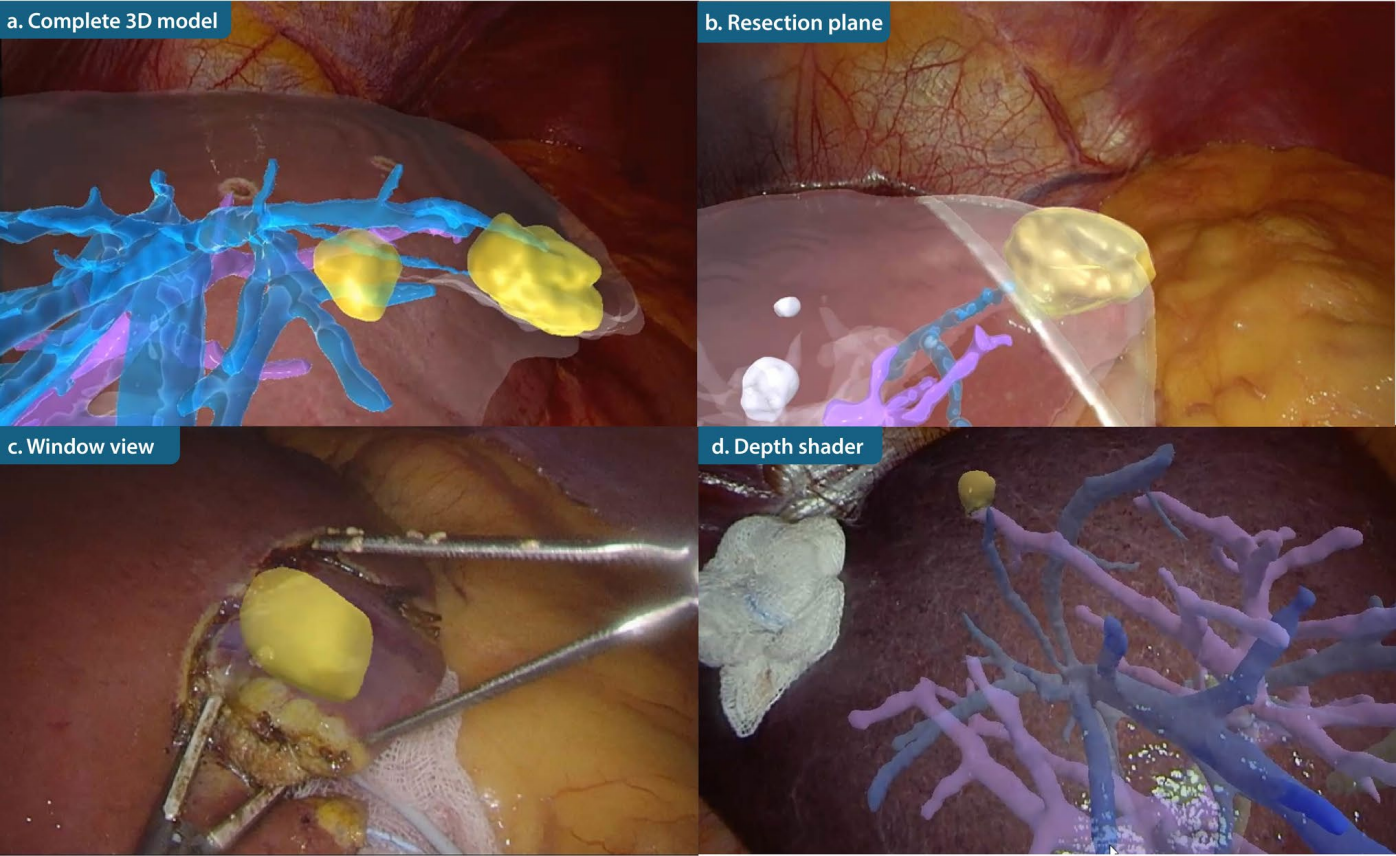
AR visualizations in selected patients: **a** complete 3D model overlay; **b** resection plane with vascular branches to segments II and III; **c** AR restricted to the central laparoscopic view; **d** reduced opacity of structures closer to the laparoscope

Fourteen patients underwent AR-guided laparoscopic liver resection. Patient characteristics and surgical outcomes can be found in Supplementary Table 1. An overview of the surgical setup is provided in Fig. 6. In all cases, attachment of the EM reference sensor to the liver surface was technically feasible and did not interfere with the surgical procedure. Sensor attachment took an average of 4 ± 1 min. In the first patient, registration could not be completed due to an incompatible calibration file for the LUS probe, which was identified only after the procedure. All subsequent cases were completed without technical errors. Across the 13 successful cases, the mean TRE and FRE were 6.3 ± 3.8 mm and 8.1 ± 1.4 mm, respectively (Table 1). The number of landmarks sampled per case ranged from 3 to 6, with vascular bifurcations being the most frequently selected structures. Registration took an average of 11 ± 7 min. AR overlays were successfully generated in all 13 cases in which registration was completed, displaying the preoperative 3D liver model including tumor(s) and vasculature within the laparoscopic video (Fig. 7). AR updated in real-time according to movements of both the laparoscope and the liver sensor. Qualitatively, accuracy remained consistent throughout the overlay time. The complete navigation setup and workflow is demonstrated in the Supplementary Video.

**Table 1:** Tumor characteristics and registration accuracy

	Tumor segment	Tumor size (mm)	*N* sampled landmarks	FRE (mm)	TRE (mm)
1	IVa	31	Technical failure		
2	IVa/b	21	3	9.1	2.6
3	III	18	5	8.1	7.5
4	IVb	12	3	7.4	9.4
5	VI	15	3	10.5	12.8
6	VI	10	4	9.0	10.3
7	V	12	3	2.5	1.8
8	II/III	62	6	8.2	6.4
9	III	25	5	5.8	8.2
10	VII	15	4	11.4	1.0
11	VI	12	5	12.2	10.3
12	II/III	41	5	6.9	2.8
13	II/III	18	4	7.2	3.3
14	III	55	4	6.8	5.9
Mean ± SD		25 ± 16	4 ± 1	6.3 ± 3.8	8.1 ± 1.4

## Discussion

This study demonstrates the feasibility of AR navigation using EM tracking in laparoscopic liver surgery. EM tracking of the laparoscope enabled stable and accurate AR visualization, and it also provided new possibilities such as tracking of the liver surface and flexible instruments such as the LUS probe. No safety concerns attributable to this technology were observed, and perioperative outcomes were comparable to those previously reported for laparoscopic liver resection [3, 23]. In this study, average target registration accuracy was 6.3 ± 3.8 mm.

A recent review reports that the accuracy of laparoscopic liver navigation systems typically ranges between 8 and 15 mm, which is consistent with the results of this study [6]. By sampling subsurface landmarks (i.e., vessel bifurcations or cysts) using a tracked LUS probe, landmarks in the proximity of the targeted lesion can be sampled, thereby achieving a system accuracy within published range. The average registration accuracy achieved in this study is comparable with our previous findings in open liver surgery [17], indicating that EM-based navigation can be successfully translated into the minimally invasive setting. For clinical tasks such as initial anatomical orientation, tumor localization, and planning of resection planes, this level of accuracy is generally considered sufficient, whereas higher accuracy would be required for continuous guidance during parenchymal transection.

Several AR views were tested in this study. Surgeons preferred views highlighting the tumor and resection plane, complemented with depth shading to improve 3D perception. While the window view was not preferred in our ex vivo validation, this contrasts with the clinical study by Ramalinho et al. [8], where a comparable visualization was preferred. This difference is likely explained by variations in surgical context and intended use of AR. In our implementation, AR was primarily used to support initial anatomical orientation and tumor localization, for which preservation of a broader anatomical context was considered important. In contrast, window-based visualizations may be more suitable for focused tasks in a stable surgical field. These findings indicate that the optimal AR configuration requires a balance between focus on target anatomy and preservation of global anatomical context.

In this study, AR was implemented using EM tracking. Selection of a tracking technology should be guided by the clinical application, as no single approach is universally optimal. In laparoscopic liver surgery, EM tracking is particularly suitable because it does not rely on line-of-sight, enabling tracking of the distal end of laparoscopic instruments within the abdominal cavity as well as tracking of the liver surface. Limitations of EM tracking include the use of wired sensors, which may introduce practical constraints in the surgical workflow, and sensitivity to ferromagnetic interference, which may affect positional accuracy. However, prior work from our group has demonstrated minimal EM interference from surrounding surgical equipment in a clinical environment [24].

While tracking of the liver surface can enhance navigation accuracy during surgery by compensating for respiratory motion and local movement caused by surgical manipulation, system accuracy typically deteriorates as the procedure progresses. Especially during major resections, the organ shape and position significantly change during the procedure and currently there are no systems capable of accurately deforming the preoperative 3D model accordingly. Consequently, accuracy data presented in this study reflects the system’s performance during the initial localization and planning phase. Obtaining a reliable ‘ground truth’ measurement for TRE during later stages was technically unfeasible due to tissue deformation and removal. Therefore, the assessment of accuracy during deep parenchymal transection remained observational. As a result, significant liver mobilization and parenchymal transection represent a fundamental limitation for the current system, as well as for AR navigation approaches more broadly. Therefore, navigation in laparoscopic liver surgery should be regarded primarily as an aid for initial anatomical orientation rather than as a precise guidance tool throughout the resection phase.

A limitation of the proposed workflow is the need for multiple integrated technical components and additional intraoperative steps, which may limit rapid adoption outside specialized centers. The relatively short setup and processing times in this study should be interpreted in the context of a dedicated team with prior experience in image-guided liver surgery and EM tracking. In addition, reliable landmark-based registration using intraoperative ultrasound requires considerable experience and training. The transition from open to laparoscopic surgery also presented consideration for the registration process compared with open surgery. Whereas hepatic vein bifurcations are routinely used as reliable landmarks in open procedures, their identification on ultrasound proved more demanding laparoscopically, partly due to vessel collapse associated with low venous pressure and pneumoperitoneum. In these cases, lowering intra-abdominal pressure improved visualization of vascular landmarks.

Although AR has the potential to mitigate the challenges of depth perception and spatial orientation inherent to minimally invasive liver surgery, intuitive visualization of subsurface anatomy remains difficult. The projection of 3D models onto a 2D laparoscopic image does not render an intuitive perception of depth and spatial relationships, particularly when lesions or vascular structures are deeply situated within the liver parenchyma. Moreover, current AR overlays are projected over surgical instruments, which may limit usability during resection. The integration of instrument tracking or real-time instrument segmentation from laparoscopic video could allow subtraction of instruments from the AR view, improving depth perception [25]. Implementation of such AI-based approaches was beyond the scope of the present study.

In addition, future work should focus on expanding the clinical cohort to demonstrate the broader benefits of this technology, as this small feasibility study AR was primarily tested in anatomical and wedge resections. Routine integration of AR into clinical workflows can only be justified if it provides a clear clinical benefit, such as improved intraoperative orientation, enhanced tumor localization, or support for surgical planning in anatomically complex cases. With increasing familiarity among surgeons and refinement of visualization strategies, AR has the potential to provide added value in more challenging scenarios, including multiple or small lesions, major resections, and tumors in close relation to major vessels. Integration with robotic platforms, may further strengthen this approach, as robotic surgery addresses inherent limitations of laparoscopy by improving surgical dexterity and enhancing depth perception with stereoscopic imaging.

In conclusion, this study demonstrates the feasibility of EM-tracked augmented reality for laparoscopic liver surgery, incorporating continuous liver motion tracking. By improving intraoperative localization of tumors and critical surrounding structures, this approach has the potential to enhance surgical insight.

## Supplementary Information

Below is the link to the electronic supplementary material.

Supplementary file1 (DOCX 15 KB)

Supplementary file2 (MP4 388212 KB)
