# Supplementary material for: Toward augmented reality in laparoscopic liver surgery using electromagnetic tracking: a clinical feasibility study
Source: Surg Endosc. 2026 Mar 18;40(4):3538–47. doi: 10.1007/s00464-026-12741-5 (PMC13053389; doi:10.1007/s00464-026-12741-5)
Supplement: Supplementary file 1 — Supplementary file1 (DOCX 15 KB) [file 464_2026_12741_MOESM1_ESM.docx]

Supplementary Material

*Patient characteristics and surgical outcomes*

Fourteen patients underwent AR-guided laparoscopic liver resection (Supplementary Table 1). Median age was 57 (range 42-78), and 8 patients (57.1%) were female. Median number of tumors per patient was 1 (range 1-4) and median tumor size of navigated lesions was 18 mm (range 10-62). Median operative time was 147 min (range 82-232 min). Major complications (Clavien-Dindo ≥III) occurred in 1/14 (7.1%), as one patient developed an intra-abdominal abscess requiring percutaneous drainage. Median length of hospital stay was 1 day (range 1-11). Resection margins were negative in all cases (R0).

**Supplementary Table 1:** Patient characteristics and surgical outcomes of laparoscopic liver resection.

|  | **Pathology** | **N tumors** | **Tumor segment^1^** | **Tumor size (mm)^1^** | **Operative time (min)** | **Conversion to open (y/n)** | **LOS (days)** | **R0 resection (y/n)** |
| --- | --- | --- | --- | --- | --- | --- | --- | --- |
| 1 | CRLM | 1 | IVa | 31 | 164 | n | 1 | y |
| 2 | NSCLC | 1 | IVa/b | 21 | 232 | n | 1 | y |
| 3 | Leiomyosarcoma | 2 | III | 18 | 211 | n | 2 | y |
| 4 | BRC | 2 | IVb | 12 | 205 | y | 4 | y |
| 5 | Melanoma | 1 | VI | 15 | 82 | n | 3 | y |
| 6 | CRLM | 2 | VI | 10 | 143 | n | 1 | y |
| 7 | NET | 2 | V | 12 | 170 | n | 1 | y |
| 8 | BRC | 1 | II/III | 62 | 127 | n | 3 | y |
| 9 | CRLM | 1 | III | 25 | 113 | n | 1 | y |
| 10 | CRLM | 1 | VII | 15 | 111 | n | 1 | y |
| 11 | CRLM | 2 | VI | 12 | 125 | n | 1 | y |
| 12 | CRLM | 1 | II/III | 41 | 151 | n | 1 | y |
| 13 | BRC | 4 | II/III | 18 | 151 | n | 3 | y |
| 14 | CCA | 1 | III | 55 | 110 | n | 11 | y |

*CRLM* colorectal liver metastasis, *NSCLC* non-small cell lung cancer, *BRC* breast cancer, *NET* neuroendocrine tumor, *CCA* cholangiocarcinoma, *LOS* length of hospital stay

^1^ Size and segment of navigated tumor.
